# Crystal structure determination as part of an ongoing undergraduate organic laboratory project: 5-[(*E*)-styr­yl]-1,3,4-oxa­thia­zol-2-one

**DOI:** 10.1107/S2056989017011264

**Published:** 2017-08-04

**Authors:** Trevor R. Nason, Melbourne J. Schriver, Arthur D. Hendsbee, Jason D. Masuda

**Affiliations:** a Department of Chemistry, Crandall University, PO Box 6004, Moncton, New Brunswick, Canada; b The Atlantic Centre for Green Chemistry and the Department of Chemistry, Saint Mary’s University, Halifax, Nova Scotia, Canada

**Keywords:** crystal structure, oxa­thia­zolone, nitrile sulfide, styr­yl, cinnamamide, conjugations

## Abstract

The title compound, C_10_H_7_NO_2_S, provides the first structure of an α-alkenyl oxa­thia­zolone ring. The phenyl ring and the oxa­thia­zolone groups make dihedral angles of 0.3 (3) and −2.8 (3)°, respectively, with the plane of the central alkene group; the dihedral angle between the rings is 2.68 (8)°. A careful consideration of bond lengths provides insight into the electronic structure and reactivity of the title compound. In the crystal, extended π-stacking is observed parallel to the *a*-axis direction, consisting of cofacial head-to-tail dimeric units [centroid–centroid distance of 3.6191 (11) Å]. These dimeric units are separated by a slightly longer centroid–centroid distance of 3.8383 (12) Å, generating infinite stacks of mol­ecules.

## Abstract 2

The title compound provides the first crystal structure of an α-alkenyl oxa­thia­zolone ring.

## Chemical context   

A common feature of the undergraduate organic chemistry teaching laboratory is the capstone, multi-step synthetic project that allows the student to integrate their lecture and laboratory experiences and capture a glimpse of research chemistry (Christiansen *et al.*, 2014 ▸). The selection of an appropriate project target compound requires reliable syntheses coupled to definitive characterizations and, if possible, real-world applications. In our teaching laboratory, we have focused our student projects on the preparation, characterization and chemistry of oxa­thia­zolone derivatives. This project compliments the lecture sections on carbonyl and heterocycle chemistry at the end of a one semester introductory organic chemistry course. The preparations of oxa­thia­zolone derivatives use methods developed in earlier laboratories and allow for subsequent chemistry of either the heterocycle or substituent group, which can be explored in a three-to-four week project cycle individually, in groups and in inquiry-based class projects. The existing literature on the oxa­thia­zolone heterocycle is sufficient but not overwhelming for the research purposes of the students. In addition, the area has been the subject of several comprehensive reviews and theses (Paton, 1989 ▸; Wentrup & Kambouris, 1991 ▸).
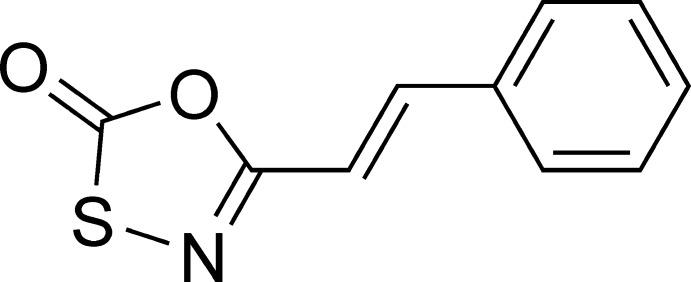


Derivatives of the oxa­thia­zolone heterocycle have been known since their first preparation in 1967 by Muhlbauer and Weiss (Muhlbauer & Weiss, 1967 ▸). Until recently, the predominant chemistry of the heterocycle was the thermal cyclo­reversion to the short-lived nitrile sulfide, a propargyl allenyl 1,3-dipole which could be trapped by electron-deficient π-bonds in reasonable yield to give families of new heterocycles (Paton, 1989 ▸). Industrially, various derivatives of the oxa­thia­zolone heterocycle have been reported as potential fungicides (Klaus *et al.*, 1965 ▸), pesticides (Hölzl & Schnyder, 2004 ▸), polymer additives (Crosby 1978 ▸) and pharmaceuticals (Russo *et al.*, 2015 ▸). In 2009, inter­est was renewed in the ring system with the report of the use of oxa­thia­zolone derivatives as selective inhibitors for mycobacterial proteasomes (Lin *et al.*, 2009 ▸). Subsequent research has uncovered potential use of styryl-substituted oxa­thia­zolone derivatives as anti­tubercular ‘warheads’ (Russo *et al.*, 2015 ▸). The significance of the structure and chemistry of styryl-substituted oxa­thia­zolone mol­ecules, especially with respect to their inter­molecular inter­actions with the proteasome, has therefore placed some significance on the structure of the title compound.

The title compound was first prepared from cinnamyl amide by Paton and coworkers (Paton *et al.*, 1983 ▸) and the synthesis was subsequently reported in a patent for use as modulating agents for amino acid receptors (Cosford *et al.*, 2005 ▸). Our inter­est in this derivative of the oxa­thia­zolone heterocycle was initially focused on the potential for exploring the chemistry of the alkene moiety in the styryl group for subsequent assessment of the substituent effect of the oxa­thia­zolone on the alkene addition and electrophilic substitution chemistry.

## Structural commentary   

The influence of oxa­thia­zolone and substituent π-conjugation on the bonding in the heterocycle has been shown spectroscopically (Markgraf *et al.*, 2007 ▸) and crystallographically (Krayushkin *et al.*, 2010*a* ▸; Krayushkin *et al.*, 2010*b* ▸) to isolate the C=N π-system from the nascent O=C=O π-system foreshadowing the facile deca­rboxylation to the nitrile sulfide. Thus the asymmetry of the C—O bonds within the heterocycle has been linked to the ease of nitrile sulfide generation. It has been proposed that the ease of deca­rboxylation is related to the length of the C1—O2 bond.

**Figure 1:**
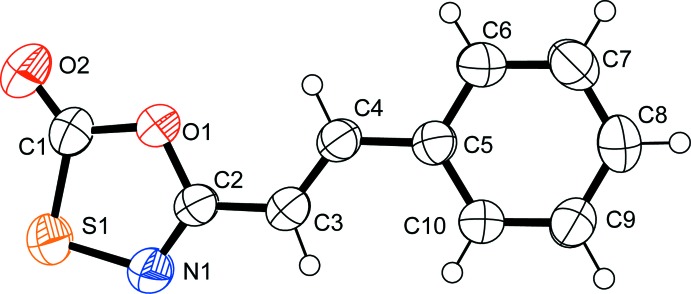
The mol­ecular structure of the title compound, showing 50% probability displacement ellipsoids.

The title compound (Fig. 1 ▸) is the first oxa­thia­zolone X-ray structure of the heterocycle substituted directly to an alkene. The C—O bonds within the heterocycle [C1—O1 = 1.3852 (19), C2—O1 = 1.3678 (17) Å] are asymmetric as expected with conjugation between the alkene and the heterocycle. The bond distances and angles are consistent with the known oxa­thia­zolone derivatives that feature a C*sp* ^2^—C*sp* ^2^ bond between the heterocycle and the unsaturated organic substituent. The C1—O1 bond [1.3852 (19) Å] is close to the statistical average for mol­ecules of this type (1.40±0.03 Å) and significantly longer than the average for mol­ecules that feature a C*sp* ^2^—C*sp* ^3^ bond between the heterocycle and the saturated organic substituent (1.374±0.005 Å). In addition, the C1—S1 bond [1.7379 (18) Å] is slightly shorter than the statistical average for mol­ecules of this type (1.75±0.02 Å). Thus the pattern of bonding within the heterocycle is consistent with the Krayuskin conjugation model, leading to the hypothesis that deca­rbonylation of this derivative should occur with milder conditions than observed for heterocycles substituted with saturated substituents.

The atoms in the ring of the oxa­thia­zolone heterocycle form bond angles that sum to 540.0° consistent with a planar ring (ideal 540°). The torsion angles O1—C2—C3—C4 [−2.8 (3)°] and C3—C4—C5—C6 [−179.81 (17)°] confirm a near planarity of the mol­ecule favorable for conjugation between the π-systems of the two rings and the central alkene.

The planarity, bond lengths and angles in the styryl fragment are comparable with previously reported values (Nilofar Nissa *et al.*, 2002 ▸; Iwamoto *et al.*, 1989 ▸). The widening of the C3—C4—C5 angle to 125.68 (14)° has been previously attributed to intra­molecular repulsion between C3 and C6 (Subramanian *et al.*, 1999 ▸). The C2—C3 distance [1.443 (2) Å] is shorter than observed in cinnamyl derivatives (Nilofar Nissa *et al.*, 2002 ▸), consistent with π-delocalization between the alkene and the heterocycle.

## Supra­molecular features   

**Figure 2:**
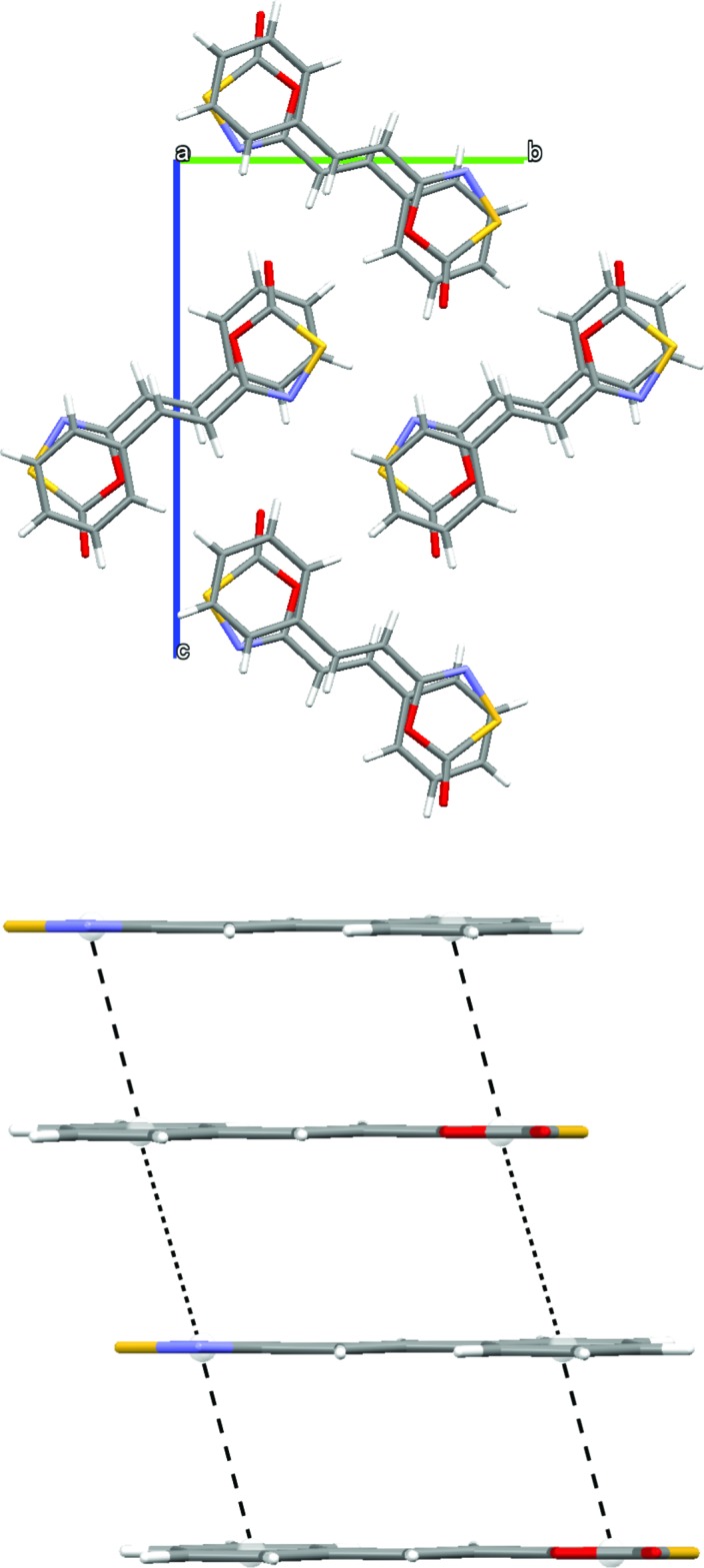
The packing diagram of the title compound showing π–π stacking parallel to the *a*-axis direction (top). Cofacial head-to-tail dimeric units [separated by long dashes, centroid–centroid distance of 3.6191 (11) Å] separated by an inter-dimer distance of 3.8383 (12) Å (small dashes, bottom).

Extended π-stacking is observed parallel to the *a*-axis direction (Fig. 2 ▸, top), consisting of cofacial head-to-tail dimeric units [centroid–centroid distance of 3.6191 (11) Å]. These dimeric units are separated by a slightly longer centroid–centroid distance of 3.8383 (12) Å (Fig. 2 ▸, bottom). It should be noted, however, that the inter­molecular S⋯N distances [3.6879 (16) Å], are significantly longer than those observed in other related S⋯N heterocyclic mol­ecules (Bridson *et al.*, 1995 ▸).

## Database survey   

There are eleven crystal structures of oxa­thia­zolone derivatives reported in the literature (Schriver & Zaworotko, 1995 ▸; Bridson *et al.* 1994 ▸, 1995 ▸; Vorontsova *et al.*, 1996 ▸; McMillan *et al.*, 2006 ▸; Krayushkin *et al.*, 2010*a* ▸,*b* ▸), which have been partially reviewed (Krayushkin *et al.*, 2010*a* ▸,*b* ▸). The structures fall into two groups: those that feature a C*sp* ^2^—C*sp* ^3^ bond between the heterocycle and the saturated organic substituent and those that feature a C*sp* ^2^—C*sp* ^2^ bond between the heterocycle and the unsaturated organic substituent (either a phenyl group or a heterocyclic ring).

## Synthesis and crystallization   

The title compound was prepared following literature methods (Cosford *et al.*, 2005 ▸) and was crystallized as large needles from a hot solution in chloro­form by cooling to room temperature followed by slow evaporation to a crystalline solid. The identity and purity of the product was determined by comparison with the literature (Paton *et al.*, 1983 ▸; Cosford *et al.*, 2005 ▸) and by UV–visible spectroscopy (CH_2_Cl_2_) λ_max_ (log ∊) : 228 nm (4.21), 307 nm (4.52).

## Refinement   

Crystal data, data collection and structure refinement details are summarized in Table 1 ▸.

## Supplementary Material

Crystal structure: contains datablock(s) I. DOI: 10.1107/S2056989017011264/hb7694sup1.cif

Structure factors: contains datablock(s) I. DOI: 10.1107/S2056989017011264/hb7694Isup2.hkl

Supporting information file. DOI: 10.1107/S2056989017011264/hb7694Isup3.cml

CCDC reference: 1565845

Additional supporting information:  crystallographic information; 3D view; checkCIF report

## Figures and Tables

**Table 1:** Experimental details

Crystal data	
Chemical formula	C_10_H_7_NO_2_S
*M*_r_	205.23
Crystal system, space group	Monoclinic, *P*2_1_/*n*
Temperature (K)	296
*a*, *b*, *c* (Å)	7.3948 (11), 9.4609 (13), 13.5183 (19)
β (°)	95.771 (2)
*V* (Å^3^)	941.0 (2)
*Z*	4
Radiation type	Mo *K*α
μ (mm^−1^)	0.31
Crystal size (mm)	0.46 × 0.21 × 0.15

Data collection	
Diffractometer	Bruker APEXII CCD
Absorption correction	Multi-scan (*SADABS*; Bruker, 2008 ▸)
*T*_min_, *T*_max_	0.637, 0.746
No. of measured, independent and observed [*I* > 2σ(*I*)] reflections	7073, 2046, 1730
*R*_int_	0.014
(sin θ/λ)_max_ (Å^−1^)	0.639

Refinement	
*R*[*F* ^2^ > 2σ(*F* ^2^)], *wR*(*F* ^2^), *S*	0.040, 0.107, 1.07
No. of reflections	2046
No. of parameters	155
H-atom treatment	All H-atom parameters refined
Δρ_max_, Δρ_min_ (e Å^−3^)	0.40, −0.18

Computer programs: *APEX2* and *SAINT* (Bruker, 2009 ▸), *SHELXS2016* (Sheldrick, 2008), *SHELXL2016* (Sheldrick, 2015 ▸) and *OLEX2* (Dolomanov *et al.*, 2009 ▸).

## supplementary crystallographic information

### Crystal data

**Table:**

C_10_H_7_NO_2_S	*F*(000) = 424
*M**_r_* = 205.23	*D*_x_ = 1.449 Mg m^−^^3^
Monoclinic, *P*2_1_/*n*	Mo *K*α radiation, λ = 0.71073 Å
*a* = 7.3948 (11) Å	Cell parameters from 4030 reflections
*b* = 9.4609 (13) Å	θ = 2.6–28.6°
*c* = 13.5183 (19) Å	µ = 0.31 mm^−^^1^
β = 95.771 (2)°	*T* = 296 K
*V* = 941.0 (2) Å^3^	Needle, pink
*Z* = 4	0.46 × 0.21 × 0.15 mm

### Data collection

**Table:**

Bruker APEXII CCD diffractometer	1730 reflections with *I* > 2σ(*I*)
Graphite monochromator	*R*_int_ = 0.014
φ and ω scans	θ_max_ = 27.0°, θ_min_ = 2.6°
Absorption correction: multi-scan (SADABS; Bruker, 2008)	*h* = −9→7
*T*_min_ = 0.637, *T*_max_ = 0.746	*k* = −12→12
7073 measured reflections	*l* = −17→17
2046 independent reflections	

### Refinement

**Table:**

Refinement on *F*^2^	0 restraints
Least-squares matrix: full	Hydrogen site location: difference Fourier map
*R*[*F*^2^ > 2σ(*F*^2^)] = 0.040	All H-atom parameters refined
*wR*(*F*^2^) = 0.107	*w* = 1/[σ^2^(*F*_o_^2^) + (0.0533*P*)^2^ + 0.2083*P*] where *P* = (*F*_o_^2^ + 2*F*_c_^2^)/3
*S* = 1.07	(Δ/σ)_max_ < 0.001
2046 reflections	Δρ_max_ = 0.40 e Å^−^^3^
155 parameters	Δρ_min_ = −0.18 e Å^−^^3^

### Special details

**Table:**

Geometry. All esds (except the esd in the dihedral angle between two l.s. planes) are estimated using the full covariance matrix. The cell esds are taken into account individually in the estimation of esds in distances, angles and torsion angles; correlations between esds in cell parameters are only used when they are defined by crystal symmetry. An approximate (isotropic) treatment of cell esds is used for estimating esds involving l.s. planes.

### Fractional atomic coordinates and isotropic or equivalent isotropic displacement parameters (Å^2^)

**Table:**

	*x*	*y*	*z*	*U*_iso_*/*U*_eq_	
S1	0.08082 (8)	1.41478 (5)	0.37424 (4)	0.0704 (2)	
O1	0.15697 (16)	1.15722 (11)	0.35689 (7)	0.0552 (3)	
O2	0.0742 (2)	1.26055 (17)	0.20881 (10)	0.0880 (5)	
N1	0.1444 (2)	1.32282 (16)	0.47790 (10)	0.0662 (4)	
C1	0.1007 (2)	1.27047 (19)	0.29653 (12)	0.0590 (4)	
C2	0.1790 (2)	1.19553 (16)	0.45493 (11)	0.0492 (3)	
C3	0.2399 (2)	1.08768 (17)	0.52613 (11)	0.0519 (4)	
H3	0.262 (3)	1.122 (2)	0.5907 (14)	0.068 (5)*	
C4	0.2691 (2)	0.95350 (17)	0.50461 (11)	0.0490 (3)	
H4	0.250 (3)	0.9270 (18)	0.4370 (15)	0.065 (5)*	
C5	0.3301 (2)	0.84338 (15)	0.57630 (10)	0.0465 (3)	
C6	0.3561 (3)	0.70636 (18)	0.54358 (13)	0.0614 (4)	
H6	0.333 (3)	0.689 (2)	0.4764 (16)	0.082 (6)*	
C7	0.4143 (3)	0.60037 (19)	0.60958 (15)	0.0693 (5)	
H7	0.430 (3)	0.509 (2)	0.5866 (16)	0.082 (6)*	
C8	0.4481 (3)	0.6292 (2)	0.70893 (14)	0.0628 (4)	
H8	0.485 (3)	0.559 (2)	0.7506 (15)	0.070 (6)*	
C9	0.4223 (3)	0.7643 (2)	0.74363 (13)	0.0644 (5)	
H9	0.446 (3)	0.786 (2)	0.8132 (16)	0.077 (6)*	
C10	0.3636 (2)	0.87006 (18)	0.67792 (12)	0.0567 (4)	
H10	0.347 (3)	0.958 (2)	0.7007 (14)	0.068 (5)*	

### Atomic displacement parameters (Å^2^)

**Table:**

	*U*^11^	*U*^22^	*U*^33^	*U*^12^	*U*^13^	*U*^23^
S1	0.0899 (4)	0.0566 (3)	0.0630 (3)	0.0181 (2)	0.0000 (2)	0.00692 (19)
O1	0.0711 (7)	0.0519 (6)	0.0415 (5)	0.0005 (5)	−0.0001 (5)	−0.0008 (4)
O2	0.1246 (13)	0.0903 (10)	0.0463 (7)	0.0098 (9)	−0.0056 (7)	0.0081 (7)
N1	0.0902 (11)	0.0561 (8)	0.0505 (8)	0.0147 (7)	−0.0019 (7)	−0.0012 (6)
C1	0.0655 (10)	0.0617 (10)	0.0488 (9)	0.0014 (8)	0.0009 (7)	0.0065 (7)
C2	0.0529 (8)	0.0516 (8)	0.0426 (7)	0.0002 (6)	0.0021 (6)	−0.0026 (6)
C3	0.0588 (9)	0.0546 (9)	0.0415 (7)	0.0022 (7)	0.0002 (6)	−0.0003 (6)
C4	0.0525 (8)	0.0525 (8)	0.0414 (7)	−0.0031 (6)	0.0027 (6)	−0.0010 (6)
C5	0.0465 (8)	0.0483 (8)	0.0453 (7)	−0.0044 (6)	0.0075 (6)	0.0001 (6)
C6	0.0801 (12)	0.0529 (9)	0.0522 (9)	0.0014 (8)	0.0113 (8)	−0.0050 (7)
C7	0.0875 (14)	0.0473 (9)	0.0745 (12)	0.0062 (8)	0.0148 (10)	−0.0009 (8)
C8	0.0669 (11)	0.0542 (9)	0.0670 (11)	0.0008 (8)	0.0049 (8)	0.0164 (8)
C9	0.0818 (12)	0.0600 (10)	0.0494 (9)	−0.0053 (8)	−0.0029 (8)	0.0057 (7)
C10	0.0739 (11)	0.0476 (8)	0.0477 (8)	−0.0020 (7)	0.0013 (7)	−0.0019 (6)

### Geometric parameters (Å, º)

**Table:**

S1—N1	1.6761 (14)	C5—C6	1.389 (2)
S1—C1	1.7379 (18)	C5—C10	1.394 (2)
O1—C1	1.3852 (19)	C6—H6	0.92 (2)
O1—C2	1.3678 (17)	C6—C7	1.382 (3)
O2—C1	1.186 (2)	C7—H7	0.93 (2)
N1—C2	1.276 (2)	C7—C8	1.369 (3)
C2—C3	1.443 (2)	C8—H8	0.90 (2)
C3—H3	0.930 (19)	C8—C9	1.381 (3)
C3—C4	1.325 (2)	C9—H9	0.96 (2)
C4—H4	0.944 (19)	C9—C10	1.379 (2)
C4—C5	1.463 (2)	C10—H10	0.90 (2)
			
N1—S1—C1	93.67 (8)	C10—C5—C4	122.42 (14)
C2—O1—C1	111.43 (12)	C5—C6—H6	117.6 (14)
C2—N1—S1	109.43 (11)	C7—C6—C5	121.04 (16)
O1—C1—S1	106.87 (11)	C7—C6—H6	121.3 (14)
O2—C1—S1	130.62 (15)	C6—C7—H7	120.1 (13)
O2—C1—O1	122.51 (17)	C8—C7—C6	120.22 (17)
O1—C2—C3	117.23 (13)	C8—C7—H7	119.7 (13)
N1—C2—O1	118.60 (14)	C7—C8—H8	118.8 (13)
N1—C2—C3	124.17 (14)	C7—C8—C9	119.98 (17)
C2—C3—H3	113.2 (12)	C9—C8—H8	121.2 (13)
C4—C3—C2	125.30 (14)	C8—C9—H9	121.0 (13)
C4—C3—H3	121.4 (12)	C10—C9—C8	119.87 (17)
C3—C4—H4	117.1 (11)	C10—C9—H9	119.1 (13)
C3—C4—C5	125.68 (14)	C5—C10—H10	119.1 (12)
C5—C4—H4	117.3 (11)	C9—C10—C5	121.11 (16)
C6—C5—C4	119.80 (13)	C9—C10—H10	119.8 (12)
C6—C5—C10	117.78 (15)		
			
S1—N1—C2—O1	−1.1 (2)	C2—C3—C4—C5	−179.74 (15)
S1—N1—C2—C3	179.16 (13)	C3—C4—C5—C6	−179.81 (17)
O1—C2—C3—C4	−2.8 (3)	C3—C4—C5—C10	0.3 (3)
N1—S1—C1—O1	−0.10 (13)	C4—C5—C6—C7	179.74 (17)
N1—S1—C1—O2	179.8 (2)	C4—C5—C10—C9	−179.52 (16)
N1—C2—C3—C4	176.93 (18)	C5—C6—C7—C8	−0.2 (3)
C1—S1—N1—C2	0.65 (15)	C6—C5—C10—C9	0.6 (3)
C1—O1—C2—N1	1.0 (2)	C6—C7—C8—C9	0.6 (3)
C1—O1—C2—C3	−179.19 (14)	C7—C8—C9—C10	−0.4 (3)
C2—O1—C1—S1	−0.45 (16)	C8—C9—C10—C5	−0.2 (3)
C2—O1—C1—O2	179.68 (17)	C10—C5—C6—C7	−0.4 (3)

## References

[bb1] Bridson, J. N., Copp, S. B., Schriver, M. J., Zhu, S. & Zaworotko, M. J. (1994). *Can. J. Chem.* **72**, 1143–1153.

[bb2] Bridson, J. N., Schriver, M. J. & Zhu, S. (1995). *Can. J. Chem.* **73**, 212–222.

[bb3] Bruker (2008). *SADABS*. Bruker AXS Inc., Madison, Wisconsin, USA.

[bb4] Bruker (2009). *SAINT*. Bruker AXS Inc., Madison, Wisconsin, USA.

[bb5] Christiansen, M. A., Crawford, C. L. & Mangum, C. D. (2014). *The Chemical Educator*, **19**, 28–33.

[bb6] Cosford, N. D., McDonald, I. A., Hess, S. D. & Varney, M. A. (2005). *U. S. Patent No. 6,956,049*. Washington, DC: US Patent and Trademark Office.

[bb7] Crosby, J. (1978). US Patent No. 4,067,862. Washington, DC: US Patent and Trademark Office.

[bb8] Dolomanov, O. V., Bourhis, L. J., Gildea, R. J., Howard, J. A. K. & Puschmann, H. (2009). *J. Appl. Cryst.* **42**, 339–341.

[bb9] Hölzl, W. & Schnyder, M. (2004). *U. S. Patent No. 6,689,372*. Washington, DC: US Patent and Trademark Office.

[bb10] Iwamoto, T., Kashino, S. & Haisa, M. (1989). *Acta Cryst.* C**45**, 1110–1112.

[bb11] Klaus, S., Ludwig, E. & Richard, W. (1965). US Patent No. 3,182,068. Washington, DC: U. S. Patent and Trademark Office.

[bb12] Krayushkin, M. M., Kalik, M. A. & Vorontsova, L. G. (2010*a*). *Chem. Heterocycl. Compd*, **46**, 484–489.

[bb13] Krayushkin, M. M., Kalik, M. A. & Vorontsova, L. G. (2010*b*). *Khim. Geterotsikl. Soedin.* **2010**, 610–617.

[bb14] Lin, G., Li, D., de Carvalho, L. P. S., Deng, H., Tao, H., Vogt, G., Wu, K., Schneider, J., Chidawanyika, T., Warren, J. D., Li, H. & Nathan, C. (2009). *Nature*, **461**, 621–626. 10.1038/nature08357 PMC3172082 19759536

[bb15] Markgraf, J. H., Hong, L., Richardson, D. P. & Schofield, M. H. (2007). *Magn. Reson. Chem.* **45**, 985–988. 10.1002/mrc.2082 17894426

[bb16] McMillan, K. G., Tackett, M. N., Dawson, A., Fordyce, E. & Paton, R. M. (2006). *Carbohydr. Res.* **341**, 41–48. 10.1016/j.carres.2005.09.012 16263102

[bb17] Muhlbauer, E. & Weiss, W. (1967). UK Pat. 1079348.

[bb18] Nilofar Nissa, M., Velmurugan, D., Shanmuga Sundara Raj, S., Fun, H. K., Kasinath, V. & Gopalakrishnan, G. (2002). *Cryst. Res. Technol.* **37**, 125–133.

[bb19] Paton, R. M. (1989). *Chem. Soc. Rev.* **18**, 33–52.

[bb20] Paton, R. M., Stobie, I. & Mortier, R. M. (1983). *Phosphorus Sulfur Relat. Elem.* **15**, 137–142.

[bb21] Russo, F., Gising, J., Åkerbladh, L., Roos, A. K., Naworyta, A., Mowbray, S. L., Sokolowski, A., Henderson, I., Alling, T., Bailey, M. A., Files, M., Parish, T., Karlen, A. & Larhed, M. (2015). *ChemistryOpen*, **4**, 342–362. 10.1002/open.201500001 PMC4522185 26246997

[bb22] Schriver, M. J. & Zaworotko, M. J. (1995). *J. Chem. Crystallogr.* **25**, 25–28.

[bb23] Sheldrick, G. M. (2015). *Acta Cryst.* C**71**, 3–8.

[bb24] Subramanian, E., Renganayaki, S., Shanmuga Sundara Raj, S. & Fun, H.-K. (1999). *Acta Cryst.* C**55**, 764–766.

[bb25] Vorontsova, L. G., Kurella, M. G., Kalik, M. A. & Krayushkin, M. M. (1996). *Crystallogr. Rep.* **41**, 362–364.

[bb26] Wentrup, C. & Kambouris, P. (1991). *Chem. Rev.* **91**, 363–373.

